# Scalable Clustering of High-Dimensional Data Technique Using SPCM with Ant Colony Optimization Intelligence

**DOI:** 10.1155/2015/107650

**Published:** 2015-10-01

**Authors:** Thenmozhi Srinivasan, Balasubramanie Palanisamy

**Affiliations:** ^1^Department of Computer Applications, Gnanamani College of Technology, AK Samuthiram, Pachal, Namakkal District, Tamil Nadu 637 018, India; ^2^Department of Computer Science and Engineering, Kongu Engineering College, Perundurai, Erode, Tamil Nadu 638 052, India

## Abstract

Clusters of high-dimensional data techniques are emerging, according to data noisy and poor quality challenges. This paper has been developed to cluster data using high-dimensional similarity based PCM (SPCM), with ant colony optimization intelligence which is effective in clustering nonspatial data without getting knowledge about cluster number from the user. The PCM becomes similarity based by using mountain method with it. Though this is efficient clustering, it is checked for optimization using ant colony algorithm with swarm intelligence. Thus the scalable clustering technique is obtained and the evaluation results are checked with synthetic datasets.

## 1. Introduction

Data mining deals with extracting useful information from datasets [1]. In data mining, clustering is a process which recognizes similar description (homogenized) groups of data on the basis of their size (profile). This involves a distance metric, in which the data points in each partition are similar to points in different partitions. A large number of data mining techniques to cluster the data are available. Few of them are CLARANS [2], Focused CLARANS [3], BIRCH [4], DBSCAN [5], and CURE [6]. Clustering is a technique that is required for various applications in pattern analysis, decision making, group and machine document retrieval, learning environment, pattern classification, and image segmentation. Clustering the dataset includes measures distance or similarity measure to partition the dataset, where data inside the clusters is similar to data outside the cluster.

The preprocessing steps are carried out before clustering, in which feature extraction and feature selection are major parts. This reduces the burden of clustering algorithm. The selection process is being done by eliminating the redundant ones. These techniques increase the speed of clustering algorithms and hence performance is improved [6]. Many feature selection techniques are proposed in the literature to reduce the size of the dataset. In those cases, reducing the dimensions using conventional feature selection leads to significant loss of data. In traditional clustering algorithm, the clustering algorithm follows distance function, but the distance function used by the algorithms to all aspects of equal treatment is not of equal importance. Where the selection of feature techniques reduces dimensions by removing irrelevant features they may have to eliminate many of the features associated with the presence of sporadic failure. For this a new class of projected clustering arises in this technique.

A projected clustering is also called subspace clustering [7] which has high-dimensional datasets, a unique group of data points that are correlated with different sets of dimensions, where the focus is to determine a set of attributes for each cluster. A set of relevant features shows the accuracy of the cluster, which has potential applications in e-commerce [8], a computer vision task [9]. There are various approaches proposed for projected clustering in the past. Few of them are CLIQUE, DOC, Fast DOC, PROCLUS, ORCLUS, and HARP [10]. After the clustering process in data mining, there is a need to check whether it is an optimized technique or not. There are various optimization techniques in queue, in which the ant colony cluster algorithm uses the characteristics of positive feedback, which is a good convergent parallel algorithm.

## 2. Related Works

A projected hierarchical clustering algorithm called hierarchical approach with automatic relevant dimension selection (HARP) is proposed in [10]. HARP works with the assumption that if the data points are similar in high-dimensional space, they also show the same similarity in lower-dimensional space. Depending on this criterion, if clusters are similar in various numbers of dimensions, they are allowed to merge. The similarity and minimum number of similar dimensions can be controlled dynamically, without the help of parameters given by user. The advantage of HARP is that it determines automatically the dimensions for each cluster without the use of input parameters, whose values are difficult to define. HARP [11] also provides interesting results on gene expression data.

The efficiency of DOC/FastDOC has been studied using FPC in [12] and has proved that it is much faster than the previous method, which also has some drawbacks, such as FPC that fits well when the cluster is in the form of a hypercube with parameter values clearly specified. A type of density based clustering algorithm for projected clustering is proposed in [13], which uses histogram construction known as efficient projective clustering by histograms (EPCH). Dense regions are spotted in each histogram by lowering threshold iteratively; for each data point corresponding to a region in a subspace a signature is generated. By identifying signatures for a large number of data points [13], projected clusters are uncovered. EPCH is a type of compression based clustering algorithm; it is faster and can handle irregular clusters, but in full-dimensional space it cannot compute distance between data points. Fuzzy clustering method (FCM) [14] is based on partition. FCM's main disadvantage is that it features the request for the number of clusters to be generated. In addition, when the data affected by noise is high, it can lead to clusters of poor quality because FCM is highly sensitive to outliers. Prediction in mobile mining for location based services to determine precious information is studied by Venkatesh et al. [15]. Clustering nonspatial data using similarity based PCM (SPCM) is proposed in [14].

## 3. Proposed Methodology

**Figure 1:**
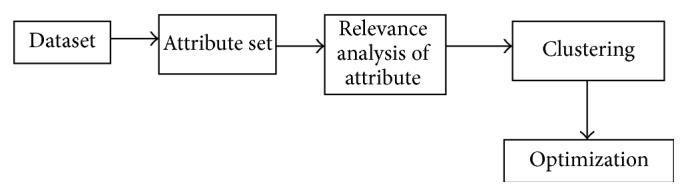
Block diagram of proposed methodology.

The proposed methodology is focused to cluster high-dimensional data in projected space. The main idea behind the development of PCM SPCM based application is to integrate with mountain cluster. SPCM has merit that it can automatically produce results clustering without requiring users to determine the number of clusters. Though this is efficient clustering, it is checked for optimization using ant colony algorithm with swarm intelligence. Thus the scalable clustering technique is obtained and the evaluation results are checked with synthetic datasets. This work is done based on the block diagram shown in Figure 1.

Let the dataset be taken as DS, having *d*-dimensional points, where attributes are denoted as *D* = {*D*_1_, *D*_2_,…, *D*_*d*_}. Then *N* data points are initialized as

(1) $$\begin{array}{c} Y=\left\{\;y_{1},y_{2},\ldots ,y_{N}\;\right\},\;where\;\;y_{i}=y_{i1},\ldots ,y_{ij},\ldots y_{id}. \end{array}$$

In this, each *y*_*ij*_  (*i* = 1,…, *N*; *j* = 1,…, *d*) corresponds to the value of *y*_*i*_ data point on *D*_*j*_ attribute.

The cluster will see a considerable number of aspects related to the above relationship in which points are close to each other in a large number. The three main steps are as follows:

- attribute relevance analysis,
- clustering,
- optimization.

### 3.1. Attribute Relevance Analysis

In attribute relevance analysis, cluster structures are displayed by identifying dense regions and their location in each dimension. For projected clustering, a cluster must contain relevant dimensions of the data in which the projection of each point of the cluster is close to a sufficient number of other projected points. The dimensions identified represent potential candidates for the clusters. Using the cluster structure, a region having high density of points is chosen compared to its surrounding regions, which represent the 1D projection of clusters. By detecting dense regions in each dimension the discrimination between dimensions that are relevant and irrelevant can be detected, and sparseness degree is then computed to detect densely populated regions in each attribute.

### 3.2. SPCM Based Clustering

After the completion of the first step, the dimension of dataset is given for reduction. It incorporates outlier elimination using sparseness estimation and similarity based possibility C-means (SPCM) algorithm. Spatial data [16] defines a location, shape, size, and orientation and it includes spatial relationships whereas nonspatial data is information which is independent of all geometric considerations. In general spatial data are multidimensional and autocorrelated and nonspatial data are one-dimensional and independent. After identification of clusters, by selecting appropriate dimensions the result is refined. The advantage of PCM is the membership function and the number of clusters is independent, and in a noisy environment with outliers it is highly vigorous [4]. When the data is a set of samples drawn from stationary processes, a framework for defining consistency of clustering algorithms is proposed in [17]. An absolute degree of data object and membership function in a cluster is assumed. There is no dependency for the membership degree on the same data objects in other clusters. So they are independent in this technique, as given in

(2) $$\begin{array}{c} U_{ip}=\frac{1}{1+{\left(d_{ip}^{2}/\eta_{i}\right)}^{1/\left(m-1\right)}}, \end{array}$$

where *u*_*ij*_ is the membership degree for object and *d*_*ij*_ is the Euclidean distance between object *x*_*j*_ and prototype *v*_*i*_. The parameter *η*_*i*_ determines the distance of membership function equals 0.5. It can be expressed as

(3) $$\begin{array}{c} \eta_{i}=P\frac{\sum_{p=1}^{N}u_{ip}^{m}d_{ip}^{2}}{\sum_{p=1}^{N}u_{ip}^{m}}, \end{array}$$

where *P* is assigned to a value of one. The prototype for inner product distance measure is as given in

(4) $$\begin{array}{c} v_{i}=\frac{\sum_{p=1}^{n}u_{ip}^{m}x_{p}}{\sum_{p=1}^{N}u_{ip}^{m}}. \end{array}$$

This algorithm follows the proceeding steps.

**Step 1 .**

A number of clusters are fixed *c*; fix 1 < *m* < *∞*; set iteration counter *l* = 1; initialize possible *c*-partition *U* ^(*O*)^; then estimate *η*_*i*_.

**Step 2 .**

Update the prototypes *V* ^(*l*+1)^ using *U* ^(*l*)^.

**Step 3 .**

Compute *U* ^(*l*+1)^ using *V* ^(*l*+1)^.

**Step 4 .**

Consider *l* = *l* + 1.

**Step 5 .**

If |*U* ^(*l*+1)^ − *U* ^(1)^| < *ε*, then process stops; else go to Step 2.

### 3.3. Mountain Method

Yager and Filev [18] proposed the mountain method which is applicable for searching approximate centers in the cluster, where the maximum of density measures are located. In Algorithm 1 one cluster is identified at a time and it is removed by eliminating density based function for other points.

In *m*-dimensional space, *R* ^*m*^, *n* data points are chosen as {*x*_1_,…, *x*_*n*_}. Let *x*_*pj*_ denote the *j*th coordinate of the *p*th point, where *p* = 1,2,…, *n* and *j* = 1,2,…, *m*. Mountain method discretizes the feature space which forms an *m*-dimensional grid in hypercube *I*_1_ × ⋯×*I*_*m*_ with nodes *N*_(*i*_1_,…,*i*_*m*_)_, where *i*_*i*_,…, *i*_*m*_ chooses values from the set [1,…, *r*_1_],…, [1,…, *r*_*m*_]. The intervals *I*_*j*_ are used to define the range of coordinates *x*_*pj*_, where the intervals *I*_*j*_ are discretized into *r*_*j*_ equidistant points.

The mountain function is calculated for the vertex point *V*_*i*_ defined as given in

(5) $$\begin{array}{c} M^{1}\left(\;V_{i}\;\right)=\overset{n}{\underset{p=1}{\sum }}e^{-\alpha d\left(x_{p},v_{i}\right)}, \end{array}$$

where *d*(*x*_*p*_, *V*_*i*_) is the distance from the data point *x*_*p*_ to the grid nodes *V*_*i*_ and *α* is the positive constant. The cluster centers are selected by the nodes having the maximum number of mountain functions. To find the cluster centers the mountain function is defined as given in

(6) $$\begin{array}{c} M^{p}\left(\;V_{i}\;\right)=M^{p-1}\left(\;V_{i}\;\right)-M_{p-1}^{\prime }\overset{n}{\underset{p=1}{\sum }}e^{-\beta d\left(x_{p-1},v_{i}\right)}, \end{array}$$

where *M*_*p*−1_′ is taken as the maximal value of mountain function and *M*_*p*−1_ and *β* are positive constants.

### 3.4. Ant Colony Optimization Based on Swarm Intelligence

The optimization algorithm is designed in such a way that probability function is used as basic pick-up and pick-down. This creates increase in similitude degree of system and maximizes the system's average similitude degree. Dorigo and Socha [19] who proposed ant colony system solve combinatorial optimization problems. Ant colony cluster [20] is motivated by the accumulation of ant bodies and ant larvae classification. The ant colony cluster algorithm uses the positive feedback characteristics. The ant conveying process is followed as the main process in the ant cluster algorithm. To convey the object to the destination, the ant considers the equivalent degree between the current object and the surrounding objects; by this way the ant will not know the other ant's location distributing load status.

Thus the ant conveying process is a simple, flexible, easy, and absolute individual behavior where objects are distributed and divided into several clusters during long time, subsequently as a concurrent process. The ant's observing radius is the main factor that influences this process, and the cluster is more efficient when radius is small [4]. Many isolated points are created, where the combination of the clusters is influenced when the observing radius is too small, which cause the inefficiency of the cluster capability. When the observing radius is large, the algorithm's convergence speed in return is expedited. The main advantage of traditional ant cluster algorithm is the adjustment of observing radius and the ant's “memory” function, and it also benefits in magnitude ameliorating aspect.

## 4. Experimental Results

The experiment is evaluated using the synthesis dataset implemented in MATLAB R2012 platform. The evaluation of this work is compared with existing method of PCKA for WDBC and MF, and the two datasets WDBC and MF are chosen because they are real and synthetic datasets. The performance results are evaluated using clustering accuracy and immunity to outliers.

Table 1 shows the clustering accuracy of the proposed and existing technique for the WDBC and MF dataset. The proposed technique achieves higher accuracy for both datasets when compared with existing PCKA technique. The reliability of PCKA technique is 90%.

**Figure 2:**
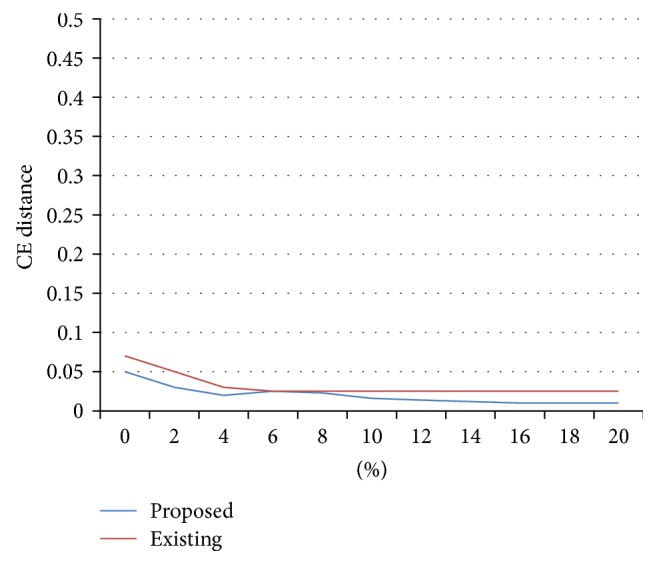
Immunity to outliers for datasets with *l*_real_ = 2 percent of *d*.

**Figure 3:**
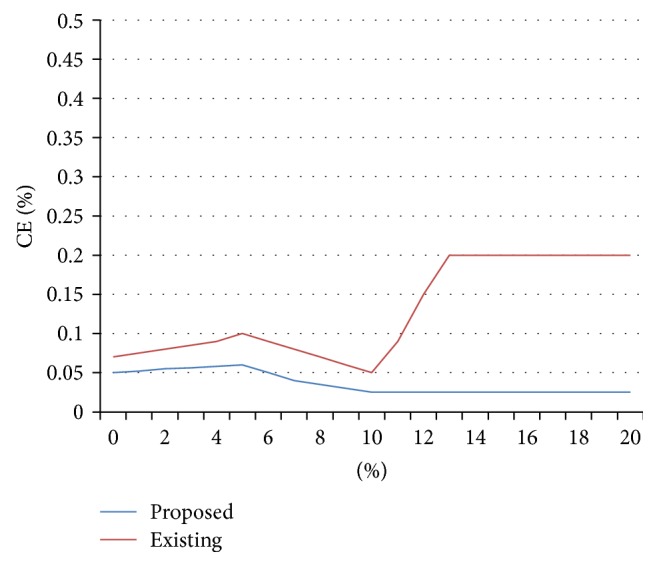
Immunity to outliers for datasets with *l*_real_ = 30 percent of *d*.

Figure 2 shows the immunity of outlier for the 2 percent of *d*, where the existing technique PCKA is affected more when compared with proposed technique and Figure 3 shows the immunity of outlier for the 30 percent of *d*, where the existing technique PCKA is affected more when compared with proposed technique.

## 5. Conclusion

An efficient high-dimensional dataset clustering is proposed in this work with its optimized results. The results were analyzed through performance evaluation based on real datasets which are synthetic. Here the SPCM method has shown its excellent performance for similarity based clustering in terms of quality, though the noisy environment dealt with outliers. This solves the problem of other fuzzy clustering methods which deals with similarity based clustering applications in the sets. This work is composed of SPCM technique which finds the clusters automatically without users input of number of clusters. And the optimized clustering result is obtained by using ant colony optimized technique with swarm intelligence. It is robust and helps to produce scalable clustering. The results given in the above section show its efficient clustering accuracy and outlier detection. The scope of further research is to deal with datasets that have a large number of dimensions.

## Figures and Tables

**Algorithm 1:**
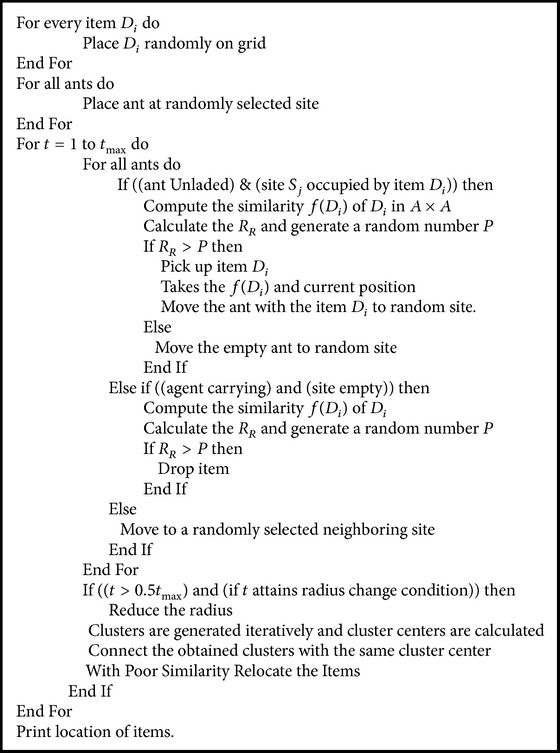
Ant colony optimization algorithm.

**Table 1:** Accuracy of clustering.

Dataset	Proposed	Existing PCKA [4]

WDBC	94.52	91.56

MF	93.7	90.45
